# Viral strain-dependent impact of plant developmental stages on the nestedness and modularity of plant-virus interaction matrices

**DOI:** 10.17912/micropub.biology.000943

**Published:** 2023-11-09

**Authors:** Izan Melero, Santiago F Elena, Rubén González

**Affiliations:** 1 Instituto de Biología Integrativa de Sistemas (CSIC - Universitat de València), Paterna, 46182 València, Spain.; 2 The Santa Fe Institute, Santa Fe, 87501 NM, USA.; 3 Institut de Biologie de l’École Normale Supérieure-CNRS-INSERM, 75005 Paris, France.

## Abstract

This study examines the specificity of adaptation of lineages of turnip mosaic virus that were experimentally evolved from *naïve* and preadapted strains to *Arabidopsis thaliana* plants at various plant developmental stages. We conducted a cross-infection experiment involving three plant developmental stages and assessed the progression of disease and symptoms. We found a significative interaction between the host developmental stage where the virus evolved and the host developmental stage in which the virus was tested. The analysis of the resulting interaction matrices revealed significant nestedness for viruses evolved from the *naïve* strain, but not for those originating from the preadapted one. Furthermore, there was an absence of modularity across all matrices. Our findings suggest that the past adaptation history of the ancestral strain influences its future evolution, and each plant developmental stage imposes unique selective constraints. The study highlights the complexity of host-parasite interactions and the potential influence of the host's developmental stage on viral adaptation.

**Figure 1.**
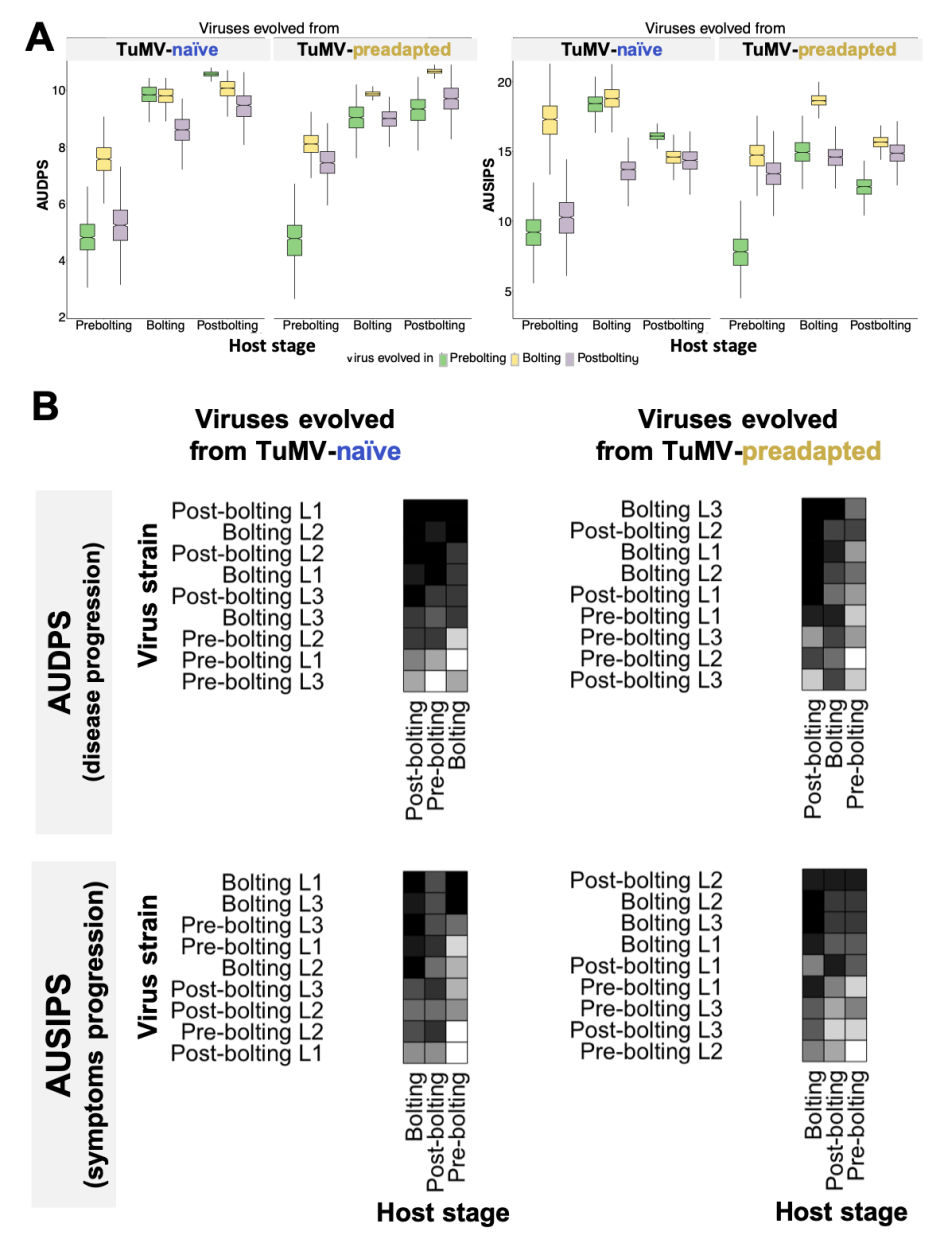
**A)** Results of the cross-infection experiment. The left panel illustrates the disease progression (area under the disease progress stairs, AUDPS), while the right panel displays symptom progression (area under the symptoms intensity progression stairs, AUSIPS). In each panel, the results are divided depending on whether the viruses evolved from an ancestral virus that was *naïve* or preadapted to *A. thaliana* . Each color represents the developmental stage at which the virus evolved, and the abscissae differentiate the developmental stage of the host where the virus was tested. **B)** Visualization of the cross-infection matrices. Each column in a matrix represents a specific plant developmental stage, while the rows represent virus strains evolved at each of the three possible developmental stages. The matrices on the left display cross-infections by viral lineages derived from the *naïve* strain, while the right-hand matrices show cross-infections by viral lineages evolved from the preadapted strain. The top row of matrices represents disease progression results, while the bottom row illustrates symptom progression. Cell shading, ranging from white to black, indicates a gradient of increasing infectivity or pathogenicity, corresponding to a value range of 0 to 9 (refer to Methods section for details).

## Description

In our previous study (Melero et al. 2023) we evolved both a *naïve* and a preadapted isolate of turnip mosaic virus (TuMV; species *Turnip mosaic virus* , genus *Potyvirus* , family *Potyviridae* ) in *Arabidopsis thaliana* Heynh Col-0 accession plants at each of three different developmental stages. An interesting question in evolutionary genetics of host-pathogen interactions is to which extent a given host or in our case, developmental stage, determines the specificity or the pathogens’ adaptation. A standard approach to study this question is to test a number of host developmental stages and virus lineages in a cross-infection experiment. In this study we performed such a cross-infection experiment involving the nine viral lineages evolved from each of the two strains. These lineages were inoculated in *A. thaliana* plants at three significant developmental stages: vegetative growth (prebolting), phase transition between vegetative and reproductive stages (bolting) and reproductive growth (postbolting). We then measured the disease progression (AUDPS; Simko and Piepho, 2012) and symptoms progression (AUSIPS; Kone et al. 2017) to generate the respective matrixes ( Fig. 1 ). AUDPS and AUSIPS collapse time-based measurements —specifically, the count of infected plants and the severity of symptoms, respectively— into single values that represent the disease’s progression, with higher values indicating faster and more severe infections. Notice that the preadapted strain was originally evolved in plants inoculated at the prebolting developmental stage.

As a first approach to scrutinize the infection matrix, we fitted the AUDPS and AUSIPS data to a MANOVA model with three orthogonal factors: ( *i* ) the evolutionary history of the evolved TuMV lineages ( *e.g* ., derived from *naïve* or preadapted isolates), ( *ii* ) the plant developmental stage ( *e.g* ., prebolting, bolting and postbolting) in which the viral lineages were evolved, and ( *iii* ) the influence of the host developmental stage at which viruses were tested. All single factors were significant ( Fig. 1A ; *P* < 0.001), with the magnitude of effect being large for the developmental stage wherein the lineages evolved (η² = 0.226) and the ancestral isolate from which the lineages were evolved (η² = 0.292). The effect was very large for the host stage in which the viral lineages were tested (η² = 0.713). Notably, there was a significative interaction with large effect between the host developmental stage where the viral lineages evolved and the host developmental stage in which they were tested ( Fig. 1A ; *P* = 0.003, η² = 0.269). A *post hoc* analysis revealed that this interaction was primarily driven by viruses evolved on the bolting stage and were inoculated into prebolting plants. Infection by these viruses on prebolting plants were more severe than those by viruses evolved during prebolting ( *P* = 0.001) or postbolting ( *P* = 0.035) stages. In other host stages, significant differences were only observed in bolting hosts inoculated with viruses that had evolved during the bolting stage; these viruses had more severe infection phenotypes compared to viruses evolved in the postbolting stage ( *P* = 0.018) ( Fig. 1A ).

As a second approach to analyze the structure of infection matrix and gather knowledge about the nature of the interaction, we used tools from networks theory. Nestedness and modularity are crucial parameters in deciphering the host-parasite interaction matrices, as they inform about the genetic mechanisms underlying the interaction between both partners (Flores et al., 2011; Moury et al., 2021) . For instance, a nested matrix should result from a gene-for-gene mechanism of interaction in which specialist and generalist host and viruses exist, while modularity would be created by a matching-allele mechanism of interaction in which related viruses only infect subsets of related hosts (Weitz et al. 2013) . Thus, we examined these features in our matrices. For the viruses evolved from the *naïve* strain, we observed significant nestedness in both disease progression ( Fig. 1B upper row, left ; *P* = 0.01) and symptoms progression ( Fig. 1B lower row, left ; *P* = 0.01). In contrast, viruses derived from the preadapted strain showed no nestedness in either of the two disease-related phenotypes ( Fig. 1B upper row, *P* ≥ 0.05 in both cases). Additionally, our modularity analysis revealed an absence of this structural feature across all four matrices.

These findings imply that when viruses evolve from a *naïve* strain, a gene-for-gene interaction mechanism with the host age shall be favored. However, this highly specific mechanism appears to be overridden when the initial strain is already adapted to a particular host age. This indicates that the ancestral strain's preliminary adaptation level can impact its interactions with the host and drive its future evolution. The lack of modularity suggests each developmental stage imposes unique selective constraints, seemingly independent of the virus' past evolutionary history. Modularity seems to be a rare occurrence in quantitative plant-parasite interactions (Moury et al., 2021) . Still, it is important to consider that the absence of modularity might also be attributed to the reduced dimensions of our matrices (9 x 3), which are derived from the limited number of relevant *A. thaliana* developmental stages.

## Methods

*Plants and growth conditions*

*A. thaliana* Col-0 were grown in a climatic chamber under a photoperiod of 16 h light (PAR of 125 µmol m ^−2^ s ^−1^ , produced by a combination of 450 nm blue and 670 nm purple LEDs in a 1:3 ratio) at 24 ºC and 8 h dark at 20 ºC, 40% relative humidity, in a mixture of 50% DSM WNR1 R73454 substrate (Kekkilä Professional, Vantaa, Finland), 25% grade 3 vermiculite and 25% 3 - 6 mm perlite. Pest management was performed by the introduction of *Stratiolaelaps scimitus* and *Steinernema feltiae* (Koppert Co., Málaga, Spain).

Plants were inoculated at three growth stages: juvenile engaged in vegetative growth (prebolting; 18 days after sowing, das), in the transition from vegetative growth to reproductive growth (bolting; 25 das), and mature developing reproductive structures (postbolting; 32 das). These stages correspond with the corresponding growth stages defined by Boyes et al. (2001): prebolting to stage 1.06, bolting to stage 5.10, and postbolting to stage 6.00.

*Virus inoculation*

We used the evolved strains of TuMV generated in Melero et al. (2023): viruses evolved in prebolting, bolting, or postbolting *A. thaliana* plants. Inoculations were done using homogenized virus-infected tissue preserved at −80 °C. The virus inoculum consisted of 100 mg of homogeneous N _2_ -frozen infected tissue mixed with 1 mL of phosphate buffer and 10% Carborundum (100 mg/mL). Plants were inoculated by rubbing 5 µL of the inoculum into three random leaves of the plant. Ten plants per developmental stage were inoculated for each virus.

*Infection characterization*

After inoculation, plants were daily inspected for visual symptoms for 14 days after the inoculation. Plants infection symptomatology was visually evaluated following a discrete scale of symptoms severity from absence of symptoms (0) to full necrosis of the plant (5) (as described in Melero et al. 2023). The infectivity and severity of symptoms data along time were used to calculate the area under the disease progress stairs (AUDPS) and intensity progression stairs (AUSIPS) values, respectively, as described in Butković et al. (2021), using the ‘agricolae’ package version 1.3-2 (de Mendiburu and Yaseen, 2020) with R version 3.6.1 (R Core Team, 2021) in RStudio version 1.3.1093.

*Statistical analysis*

We employed a multivariate analysis of variance (MANOVA) approach to discern the effects and interactions of several key factors on the two disease-related variables (AUDPS, AUSIPS). Our factors of interest included the plant developmental stage in which the viral lineages were evolved ( *E* ), the developmental stage in which the lineages were tested ( *T* ), and the ancestral virus from which the lineages were derived ( *V* ). Data was fitted to the following multivariate linear model:

**X** *_ijkl_* ~ **μ** + *E _i_* + *T _j_* + *V _k_* + ( *E* *T* ) *_ij_* + ( *E* *V* ) *_ik_* + ( *T* *V* ) *_jk_* + ( *E* *T* *V* ) *_ijk_* + **ε** *_ijkl_*

where **X** *_ijkl_* is the vector of trait values, **μ** is the vector of grand mean values, and **ε** *_ijkl_* the vector of errors assumed to be distributed. The magnitude of each factor, and their interactions was evaluated using the η ^2^ statistic. Usually, η ^2^ > 0.15 are considered as large effects (Lakens, 2013) . The model was fitted using the *manova* () function from the ‘stats’ package version 3.6.1. We determinized whether there are significant differences between the means of identified groups using Wilks' **Λ** statistic. Pairwise *post hoc* multiple comparisons were conducted using the *emmeans* () function from the ‘mmeans’ package version 1.4.7 (Lenth, 2020) . Tukey's method was used to adjust for multiple comparisons in the pairwise comparisons.

*Infection matrix analysis*

Following the methods described by Moury et al. (2021) the AUDPS and AUSIPS values were transformed into integers and the resulting matrix was analyzed for estimation of its nestedness and modularity. The original values were transformed into integers in the *i* ∈ (0, 9) interval, following ten intervals with equal sizes which bounds were

[Χ _min_ + *i* (Χ _max_ - Χ _min_ )/ 10,Χ _min_ + ( *i* + 1) (Χ _max_ - Χ _min_ )/10].The nestedness and modularity of the matrices were estimated, and their statistical significance tested respectively with the ‘bipartite’ version 2.18 and ‘igraph’ version 1.5.0.9008 packages. The nestedness was evaluated using the wNODF algorithm (Almeida-Neto et al., 2008) and the modularity using the spinglass algorithm (Newman & Girvan, 2004) . The statistical significance of the nestedness and modularity was calculated by comparing the matrices to 100 matrices generated under the B null model. This null model is a constrained one that generates matrices with identical column and row marginal sums as the actual matrix using Patefield (1981) algorithm.
